# A new twist to the emerging functions of ceramides in cancer: novel role for platelet acid sphingomyelinase in cancer metastasis

**DOI:** 10.15252/emmm.201505161

**Published:** 2015-04-09

**Authors:** Yusuf A Hannun, Benjamin Newcomb

**Affiliations:** Department of Medicine, The Stony Brook Cancer CenterStony Brook, NY, USA

## Abstract

It is now appreciated that sphingolipids constitute a rich class of bioactive molecules that include ceramide, sphingosine, and sphingosine 1-phosphate whose formation is controlled by a network of highly regulated enzymes (Hannun & Obeid, 2008). Notably, several stress stimuli induce the production of ceramide, which, as a single entity, has been traditionally associated with apoptotic and growth suppressive functions. However, recent data clearly suggest that this simplistic formulation is no longer tenable.

See also: A Carpinteiro et al (June 2015)

The actions of distinct enzymes introduce variations in the structure of ceramide (chain length, fatty acid saturation, and hydroxylation), resulting in several individual ceramides. Furthermore, the production of sphingolipids is compartmentalized within the cell and controlled by distinct enzymes (Fig 1A). Among the best studied are the sphingomyelinases (SMases), a family of at least four different gene products that show distinct subcellular localization and distinct mechanisms of regulation (i.e., they respond to different stimuli). Among them, acid SMase (ASM in humans, Asm in mice), the product of the SMPD1 gene (sphingomyelin (SM) phosphodiesterase 1), modulates SM homeostasis but also participates in the response to a diverse set of cytokines or apoptotic stimuli (Jenkins *et al*, 2010). Indeed, several tumor types are known to have low levels of ASM. However, recent findings (Petersen *et al*, 2013), including the study by Carpinteiro *et al* (2015) in this issue of *EMBO Molecular Medicine*, are challenging this notion and redefining the study of ASM in tumor biology.

**Figure 1:**
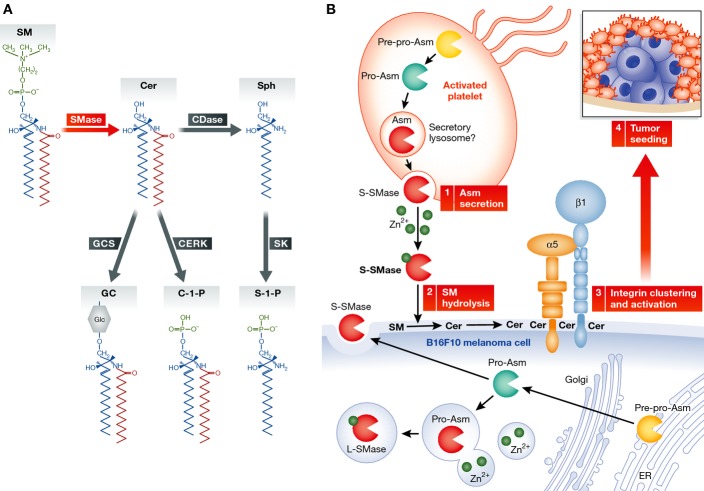
Hydrolysis of sphingomyelin on tumor cell plasma membranes, by platelet derived S-ASM, enhances tumor seeding in the lungs (A) Overview of the sphingomyelin hydrolytic pathway. (B) Platelet activation, in proximity to B16F10 cells, induces Asm secretion from platelets (1). The platelet-derived S-SMase acts on tumor cells to hydrolyze SM to Cer at the outer plasma membrane (2), resulting in integrin clustering and activation (3). Activated integrin signaling promotes tumor seeding in the lungs (4).

Under normal physiologic conditions, the SMPD1 transcript is translated into immature pre-pro-ASM that is post-translationally modified to produce secretory ASM (S-ASM) and lysosomal ASM (L-ASM). Deficiency of L-SMase results in Niemann–Pick disease types A and B (NPD A and NPD B, respectively). NPD A, with < 1% of normal ASM activity, is characterized by lipid accumulation in the lysosomes resulting in organ failure and severe neurologic deficits, whereas NPD B patients have ∽4% normal ASM activity and survive well into adulthood. S-ASM is stimulated by cytokines and mediates specific responses such as induction of chemokines, but also acts on SM in lipoproteins. Thus, even with one enzyme of sphingolipid metabolism, there could be more than one subcellular site of action. The study by Carpinteiro *et al* (2015) adds another layer by demonstrating cross-tissue action of S-ASM in the setting of tumor metastasis *in vivo*.

Carpinteiro *et al* (2015) were interested in defining the role of Asm and ceramide in hematogenous spread of tumor cells. They found that the number of lung metastases formed after injecting melanoma cancer cells into syngeneic mice was significantly decreased in the Asm knockout (KO) mice. Similar results were obtained using amitriptyline, an indirect inhibitor of Asm. These effects were not due to growth suppression once in the lung but rather to effects on earlier stages of the metastatic process. Moreover, ablation of Asm in the tumor cells had no effect on their ability to seed the lung. Together, the results suggest a role for host Asm in metastasis. In probing this role further, Carpinteiro *et al* (2015) turned to platelets presumably because of the role for platelets in the early phases of metastasis where platelet-rich thrombi form around tumor cells and create a favorable microenvironment (Fig 1B). Furthermore, treatment of platelets with thrombin induces Asm secretion (Romiti *et al*, 2000), raising the possibility for a role of Asm in platelet/tumor interactions. Indeed, the authors found that transfusion of wild-type (WT) platelets into the Asm KO restored the metastatic potential of melanoma cells, thus demonstrating that the defect in metastasis is largely due to the absence of platelet Asm.

One exciting corollary from this study relates to observations in the heterozygous Asm mouse (Asm^−/+^). Several studies have relied heavily on mouse models of NPD with total KO of the Smpd1 gene (Asm^−/−^). These mice have no detectable Asm activity, and they display many of the phenotypes seen in NPD A patients, including rapid and progressive neuropathology, accumulation of foam cells in viscera, and dramatically shortened lifespan. This severe clinical phenotype complicates the interpretation of results, even when conducted at early ages (e.g., 7 weeks in this study); e.g. foamy macrophages are present in bronchial alveolar lavage of Asm^−/−^ animals at 3–4 weeks of age (Dhami *et al*, 2001). Therefore, it becomes difficult to discern primary effects due to loss of ASM from consequences of organ failure and cell pathology, many of which, such as dysfunction of the endolysosomal system, are shared among different lysosomal storage disorders and thus do not signify specific roles for the target enzyme. This may underlie some of the conflicting results on Asm. For example, Asm-deficient animals develop more liver metastases following intra-splenic injection of colon carcinoma cells (Osawa *et al*, 2013), but it was not clear whether this was secondary to liver dysfunction or due to specific actions mediated by Asm. In this regard, there has been a paucity of studies employing the heterozygous Asm mouse as an Asm-attenuated model. Asm^−/+^ mice have 40–70% activity, and in contrast to nullizygous mice, no developmental phenotype has been observed (Horinouchi *et al*, 1995). Notably, Moles *et al* (2010) employed heterozygous animals to define a role for Asm-mediated cathepsin activation in liver fibrosis. In this context, Carpinteiro *et al* (2015) employed Asm^−/+^ mice and found significant attenuation of metastasis, strongly adding credence to a pro-metastatic role for host Asm.

Mechanistically the authors show that Zn^2+^-dependent Asm activity (a signature of S-ASM) and ceramide levels increased in the media following co-incubation of B16F10 tumor cells with platelets and that platelets are the source of the secreted Asm. Indeed, treatment of B16F10 cells with recombinant Asm or with ceramide restored tumor formation in KO mice.

Using an antibody that recognizes ceramide, the authors suggest a role for ceramide raft formation by demonstrating that ceramide and integrins co-localized on the surface of B16F10 cells after treatment with Asm or with WT platelets. The authors also show that Zn^2+^-dependent L-Asm has sufficient activity at neutral pH to alter the lipid content of the plasma membrane, confirming and extending previous work by Schissel *et al* (1996). This would constitute a mechanism for the trans-cellular action of ASM.

Several important questions are generated from this study. A critical biophysical property of proposed ceramide rafts is the threshold level at which plasma membrane ceramide begins forming rafts. From Fig 3 in Carpinteiro *et al* (2015), it appears that increasing the total ceramide content to double the physiologic concentration induces raft formation and integrin activation. However, other work has shown that plasma membrane ceramide can induce apoptosis of tumor cells. This leaves the possibility that non-apoptotic metabolites of ceramide, such as sphingosine 1-phosphate, may also play a significant role in integrin activation. This possibility is not investigated in the current study, and the conclusion that ceramide rafts induce integrin clustering is primarily based on co-localization of integrins with ceramide, the latter detected by immunostaining with anti-ceramide antibodies. However, anti-ceramide staining has not been fully validated, and ceramide antibodies are known to immunostain other lipids including sphingomyelin (Cowart *et al*, 2002). Validation of the ceramide antibody should be established (e.g., by artificial production of ceramide at the plasma membrane, with bacterial SMase, for example, or by preventing ceramide production with selective inhibitors or accelerating ceramide removal).

Since several tumor types are known to express high levels of sphingomyelin and ceramide-producing enzymes (Komori *et al*, 1999), it would be interesting to determine whether this is associated with a higher intrinsic metastatic rate and whether pharmacologic inhibition of Asm could help reduce tumor metastasis. Asm inhibition has traditionally relied on the use of tricyclic antidepressants and other cationic amphipathic drugs (CADs), lysosomotropic molecules that induce loss of ASM (but also other enzymes such as acid ceramidase) (Hurwitz *et al*, 1994). Given their lack of specificity, these reagents can be used judiciously in cell biology and in *in vivo* studies to rule out a role for ASM if the results are negative. If the results are positive, however, they can only be considered as supporting evidence. For therapeutic purposes, it is thus unlikely that this class of molecules can become a class of viable inhibitors of ASM due to both specificity and potency issues.

Moreover, the use of CADs has resulted in some conflicting results. For example, Petersen *et al* utilized c-src^*Y527F*^-transformed NIH 3T3 murine embryonic fibroblasts to demonstrate that lysosomotropic agents, which inhibit Asm, selectively kill transformed cells. Using mammary fat pad injections of MCF7 breast carcinoma cells in SCID mice, they showed reduced xenograft growth in mice treated with siramesine (Petersen *et al*, 2013). On the other hand, Osawa *et al* (2013) showed that Asm KO mice had increased hepatic colonization following splenic injection of SL4 colon carcinoma cells. As such, we believe that there is a pressing need to develop more specific and potent inhibitors of Asm and, if possible, target L-ASM selectively compared to S-ASM.

In conclusion, Carpinteiro *et al* (2015) provide compelling evidence of cross talk between platelets and tumor cells accomplished by trans-cellular signaling mediated by Zn^2+^ dependent Asm, which they show is required for lung seeding of melanoma cells. Although much still needs to be done, their work presents a new paradigm in the investigation of sphingolipids in cancer biology.

## References

[b1] Carpinteiro A, Becker KA, Japtok L, Hessler G, Keitsch S, Pozgajova M, Schmid KW, Adams C, Müller S, Kleuser B (2015). Regulation of hematogenous tumor metastasis by acid sphingomyelinase. EMBO Mol Med.

[b2] Cowart LA, Szulc Z, Bielawska A, Hannun YA (2002). Structural determinants of sphingolipid recognition by commercially available anti-ceramide antibodies. J Lipid Res.

[b13] Dhami R, He X, Gordon RE, Schuchman EH (2001). Analysis of the lung pathology and alveolar macrophage function in the acid sphingomyelinase-deficient mouse model of Niemann-Pick disease. Lab Invest.

[b3] Hannun YA, Obeid LM (2008). Principles of bioactive lipid signalling: lessons from sphingolipids. Nat Rev Mol Cell Biol.

[b4] Horinouchi K, Erlich S, Perl DP, Ferlinz K, Bisgaier CL, Sandhoff K, Desnick RJ, Stewart CL, Schuchman EH (1995). Acid sphingomyelinase deficient mice: a model of types A and B Niemann–Pick disease. Nat Genet.

[b5] Hurwitz R, Ferlinz K, Sandhoff K (1994). The tricyclic antidepressant desipramine causes proteolytic degradation of lysosomal sphingomyelinase in human fibroblasts. Biol Chem Hoppe Seyler.

[b6] Jenkins RW, Canals D, Idkowiak-Baldys J, Simbari F, Roddy P, Perry DM, Kitatani K, Luberto C, Hannun YA (2010). Regulated secretion of acid sphingomyelinase: implications for selectivity of ceramide formation. J Biol Chem.

[b7] Komori H, Ichikawa S, Hirabayashi Y, Ito M (1999). Regulation of intracellular ceramide content in B16 melanoma cells. Biological implications of ceramide glycosylation. J Biol Chem.

[b8] Moles A, Tarrats N, Morales A, Domínguez M, Bataller R, Caballería J, García-Ruiz C, Fernández-Checa JC, Marí M (2010). Acidic sphingomyelinase controls hepatic stellate cell activation and in vivo liver fibrogenesis. Am J Pathol.

[b9] Osawa Y, Suetsugu A, Matsushima-Nishiwaki R, Yasuda I, Saibara T, Moriwaki H, Seishima M, Kozawa O (2013). Liver acid sphingomyelinase inhibits growth of metastatic colon cancer. J Clin Invest.

[b10] Petersen NH, Olsen OD, Groth-Pedersen L, Ellegaard AM, Bilgin M, Redmer S, Ostenfeld MS, Ulanet D, Dovmark TH, Lønborg A (2013). Transformation-associated changes in sphingolipid metabolism sensitize cells to lysosomal cell death induced by inhibitors of acid sphingomyelinase. Cancer Cell.

[b11] Romiti E, Vasta V, Meacci E, Farnararo M, Linke T, Ferlinz K, Sandhoff K, Bruni P (2000). Characterization of sphingomyelinase activity released by thrombin-stimulated platelets. Mol Cell Biochem.

[b12] Schissel SL, Schuchman EH, Williams KJ, Tabas I (1996). Zn^2+^-stimulated sphingomyelinase is secreted by many cell types and is a product of the acid sphingomyelinase gene. J Biol Chem.

